# Injuries and Outcomes of Ground-level Falls Among Older Patients: A Retrospective Cohort Study

**DOI:** 10.5811/westjem.35281

**Published:** 2025-01-30

**Authors:** Vincent Kan, Wilson Huang, Gretta Steigauf-Regan, Jill Anderson, Ivy Dang, Chad Darling

**Affiliations:** * UMass Chan Medical School, Department of Emergency Medicine, Worcester, Massachusetts; † University of California San Francisco, Department of Emergency Medicine, San Francisco, California

## Abstract

**Study Objective:**

We sought to determine the overall rates of traumatic injuries and whether the rates of traumatic injuries and various clinical outcomes differed among older patients presenting to a tertiary-care emergency department (ED) after a ground-level fall (GLF) and who underwent whole-body computed tomography.

**Methods:**

We conducted a retrospective cohort study of patients ≥65 years of age who presented to the ED with a GLF and received a whole-body CT from January 1–December 31, 2021. Age was stratified into age groups: 65–74; 75–84; and 85+. We presented a descriptive analysis of traumatic injuries, intensive care unit (ICU) admissions, and all-cause mortality rates. We used multivariable logistic regression to determine the association between increasing age, traumatic injuries, and clinical outcomes.

**Results:**

Of 638 patients in the cohort, 120 (18.9%) sustained thoracic injuries and 80 (12.5%) sustained intracranial hemorrhages. Only five (0.8%) patients sustained an intra-abdominal injury, while 134 (21.0%) were admitted to the ICU, and 31 (4.8%) died during their index hospitalization. Head injuries (odds ratio [OR] 6.21, 95% CI 3.65–10.6, *P* < 0.001) and thoracic injuries (OR 5.25, 95% CI 3.30–8.36, *P* < 0.001) were associated with increased odds of ICU admission, whereas head injuries (OR 3.21, 95% CI 1.41–7.31, *P* < 0.01) and cervical injuries (OR 3.37, 95% CI 1.08–10.5, *P* < 0.05) were associated with increased odds of in-hospital, all-cause mortality. There were no statistically significant differences in the rates of injuries sustained between the respective age groups. There was no association between increasing age and ICU admissions or in-hospital, all-cause mortality rates.

**Conclusion:**

Among patients aged ≥65 years of age who presented to the ED after a ground-level fall and underwent whole-body CT, thoracic injuries and intracranial hemorrhages were associated with increased odds of ICU admissions. We found no significant differences in injury rates or outcomes across age groups, indicating that age alone should not guide ICU admission decisions. These findings suggest that the use of whole-body CT in this population should be selective and guided by clinical judgment rather than applied universally.

Population Health Research Capsule What do we already know about this issue?

*Falls are the leading cause of injury in older adults, yet optimal use of whole-body CT in low-energy mechanisms such as ground-level falls (GLF) is unclear.*

What was the research question?

*Do traumatic injury rates and clinical outcomes differ with age among older patients undergoing whole body CT for ground-level falls?*

What was the major finding of the study?

*Thoracic (OR 5.25, 95% CI 3.30–8.36) and head injuries (OR 6.21, 95% CI 3.65–10.6) after a ground-level fall were associated with ICU admissions. Only five (0.8%) patients sustained an intra-abdominal injury.*

How does this improve population health?

*Selective whole-body CT use in GLF patients can improve care efficiency by focusing on clinically significant injuries while reducing unnecessary imaging.*

## INTRODUCTION

### Background

Falls are the leading cause of fatal and non-fatal injuries among the elderly. Up to 40% of men and women ≥65 years of age in the community fall each year.^1^ Injuries related to falls among the elderly account for three million emergency department (ED) visits and 50 billion dollars of US healthcare spending annually.^2^ Ground-level falls (GLF), defined as falls from a standing height, are particularly common among older patients. Age-related physiologic changes create significant fall-related morbidity and mortality in this patient cohort.^3^

### Importance

Patients who present to the ED for trauma-related complaints are often evaluated with computed tomography (CT) for their accuracy and reliability in detecting injuries. The routine use of non-selective, whole-body (head to pelvis) CT is becoming an increasingly common diagnostic modality in these patients, particularly in those involved in high-energy mechanisms such as motor vehicle collisions, due to the more widespread availability of CT imaging and changing clinical practice patterns.^4^ While whole-body CT is frequently used in high-energy trauma, its application in low-energy mechanisms like GLFs remains less clear and more variable. Several studies have shown conflicting evidence as to whether whole-body CT is warranted in trauma patients.^5^ ^,^ ^6^ Given the low kinetic energy impact from GLFs, it is unclear whether the indiscriminate use of whole-body CT in GLFs can improve patients’ outcomes by detecting clinically relevant injuries.

### Goals of This Investigation

Our objectives of in this study were to determine the overall rates of traumatic injuries and clinical outcomes and whether the rates of traumatic injuries and clinical outcomes were associated with increasing age among patients ≥65 years of age presenting to a tertiary-care ED with a GLF and who underwent whole-body CT. We hypothesized that due to decreased physiological reserves and increased fragility, the incidence of traumatic injuries and adverse outcomes after a GLF among this selective population would increase with advancing age.

## METHODS

### Study Design and Setting

This study was approved by the Human Institutional Review Board. We conducted a retrospective cohort study of patients treated at this tertiary-care Level I trauma academic medical center with 90,000 annual ED visits. We adhered to the previously published methodological criteria for health record review studies.^7^

### Study Population

We identified all patients ≥65 years of age who presented to the ED with a GLF and received a whole-body CT between January 1–December 31, 2021. At our institution, a whole-body CT is defined as a CT of the head, chest, abdomen and pelvis, cervical spine, thoracic spine, and lumbar spine; it includes intravenous contrast administration to evaluate for soft tissue injury of the thorax and abdomen. A GLF is defined as falling from a standing height, chair, wheelchair, or out of bed.

### Measurements

Study variables collected included basic demographic characteristics (age and sex as identified by patient), antiplatelet or anticoagulant use, medical comorbidities, initial Glasgow Coma Scale (GCS) score, initial heart rate and systolic blood pressure, and traumatic injuries found on CT. Data was collected using a standardized data collection form through the electronic health record (Epic Systems, Verona, WI) by trained research assistants (RA) and a resident physician (WH). None of the trained RAs or the resident physician knew the study objectives. Data points collected included all acute traumatic injuries identified on the final CT imaging radiology reports. Co-author GS performed a duplicate review of 10% of the health records for interobserver reliability assessment. We used the Cohen kappa to determine the inter-rater reliability of data abstraction.

### Study Outcomes

Our primary study outcome measures included the rate of various acute traumatic injuries, admission to the intensive care unit (ICU), and all-cause, in-hospital mortality. Traumatic injuries included intracranial hemorrhages (ICH), thoracic injuries, intra-abdominal injuries, cervical spine fractures, thoracic spine fractures, and lumbar spine fractures. Intracranial hemorrhages were defined as any epidural, intraparenchymal, intraventricular, subarachnoid, or subdural hematomas or hemorrhage that were believed to be traumatic in etiology. We defined thoracic injuries as hemothoraces, pneumothoraces, pulmonary contusions, or rib fractures. Intra-abdominal injuries were defined as any solid organ or hollow viscous injuries. We excluded minor soft tissue injuries or hematomas, subacute or chronic traumatic injury findings, and non-traumatic findings on CT imaging. For our secondary outcome measures, we analyzed the association between age, traumatic injuries, and clinical outcomes, including ICU admissions and in-hospital, all-cause mortality. Age was stratified into age groups: 65–74; 75–84; and 85+.

### Data Analysis

Descriptive statistics are presented as means ± standard deviations for continuous variables, and categorical variables are reported as percentages. Incidences of traumatic injuries, ICU admissions, and all-cause mortality are reported as proportions with accompanying 95% confidence intervals (CI). Differences between our age-specific comparison groups (65–74, 75–84, 85+) were examined using ANOVA for continuous variables and chi-squared and Fisher exact tests for categorical variables. Two-tailed values of *P* < 0.05 were considered statistically significant. We performed bivariate analysis to identify variables associated with clinical outcomes. Using the 65–74 cohort as the reference group, we used multivariable logistic regression to determine the association between age, traumatic injuries, and clinical outcomes controlling for medical comorbidities, antithrombotic use, and statistically significant traumatic injuries. Data analysis was performed using STATA/MP Version 17 (StataCorp, College Station, TX).

## RESULTS

### Baseline Patient Characteristics

A total of 638 patients met our inclusion criteria during the one-year period under study. The average age of the study population was 82.1 ± 9.0 years; 60.0 % were women, and 62.9% were on at least one antithrombotic agent, with 33.7% on an antiplatelet and 39.1% on an anticoagulant. The average number of comorbidities was 1.5 ± 1.3 (Table 1).

**Table 1.** Study population characteristics according to age.

		Age (yrs)		
	65–74 (n = 159)	75–84 (n = 213)	85+ (n = 266)	*P*-value
Age, years	70.1 ± 3.0	80.1 ± 2.7	90.7 ± 4.3	-
Sex (female), %	48.4	55.4	69.6	< 0.01
SBP	136 ± 27	144 ± 29	146 ± 29	< 0.01
HR	87 ± 22	85 ± 26	81 ± 20	0.02
GCS	14 ± 3	14 ± 2	14 ± 2	^1^
Comorbidities, %				
CHF	26.4	26.7	27.1	^1^
COPD	21.4	18.8	9.02	< 0.01
CVA/TIA	26.4	21.1	16.9	^1^
Dementia	14.5	21.1	29.3	< 0.01
Diabetes	31.5	35.7	18.8	< 0.01
MI	13.8	17.4	14.3	^1^
Osteoporosis	10.7	16.9	21.1	0.02
Antithrombotic, %				
Anticoagulant	39.0	43.7	35.7	^1^
Antiplatelet	32.7	36.2	32.7	^1^

1 Not statistically significant.

*CHF*, congestive heart failure; *COPD*, chronic obstructive pulmonary disease; *CVA/TIA*, cerebrovascular accident/transient ischemic attack; *GCS*, Glasgow Coma Scale; *MI*, myocardial infarction; *HR*, heart rate; *SBP*, systolic blood pressure.

### Main Results

Among the 638 patients who sustained a GLF, 120 patients (18.9%) sustained thoracic injuries, and 80 (12.5%) sustained ICH. Sixty (9.8%) patients sustained thoracic spine injuries, 51 (8.0%) s sustained lumbar spine injuries, and 34 (5.3%) patients sustained cervical spine injuries. Only five (0.8%) patients sustained an intra-abdominal injury (Figure). Of the five patients with intra-abdominal injuries, all five were found to have pertinent physical exam findings, initial unstable vital signs (systolic blood pressure <90 or heart rate 100), or abnormal GCS (<15). There were no statistically significant differences in the rates of various injuries sustained between the respective age groups (Table 2).

**Figure.**
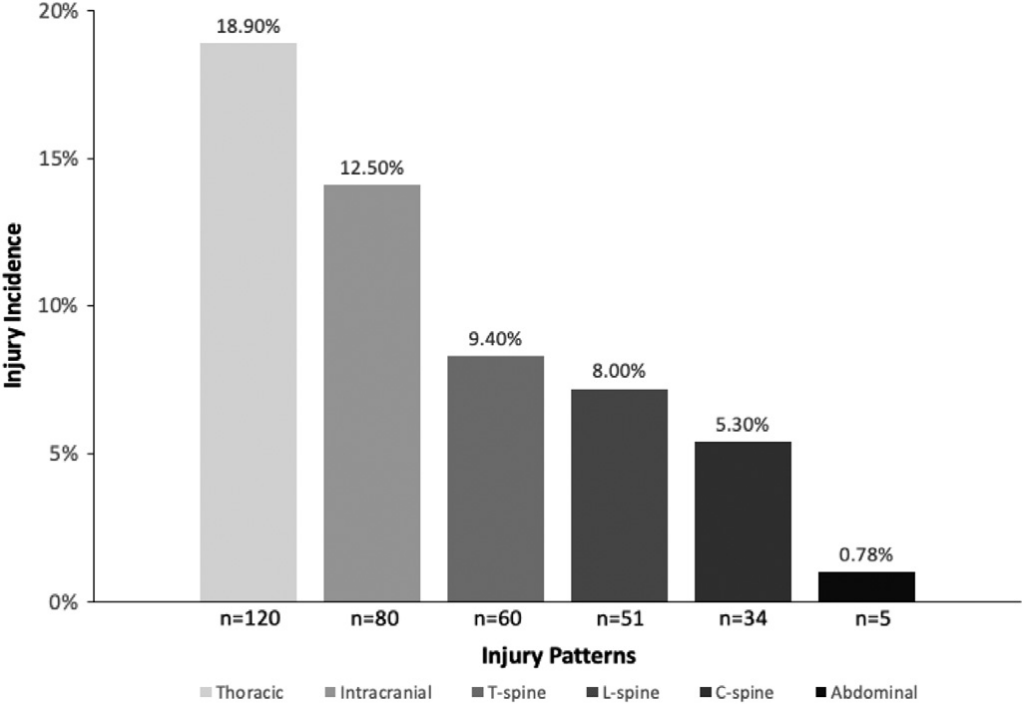
Incidence proportion among different injuries.

**Table 2.** Traumatic injuries and emergency department dispositions stratified by patient age.

	65–74 (n = 159)	75–84 (n = 213)	85+ (n = 266)
Traumatic injuries, no. (%)			
Intracranial	16 (10.1%)	32 (15.0%)	32 (12.0%)
Thoracic	32 (20.1%)	40 (18.8%)	48 (18.1%)
Intra-abdominal	1 (0.63%)	0	4 (1.50%)
Cervical spine	10 (6.29%)	15 (7.04%)	9 (3.38%)
Thoracic spine	8 (5.03%)	24 (11.3%)	28 (10.5%)
Lumbar spine	11 (6.92%)	18 (8.45%)	22 (8.27%)
ED disposition, no. (%)			
ICU	38 (23.9%)	45 (21.1%)	51 (19.2%)
Death	2 (1.26%)	5 (2.35%)	3 (1.13%)

No statistical significance between all age groups.

*ED*, emergency department; *ICU*, intensive care unit.

A total of 134 (21.0%) patients were admitted to the ICU, and 31 (4.8%) died during their index hospitalization. There were no statistically significant differences in the rates of clinical outcomes between the respective age groups. Using multivariable logistic regression models, we found no association between increasing age and ICU admissions or in-hospital, all-cause mortality rate (Table 3). In contrast to age, head injuries (odds ratio [OR] 6.15, 95% CI 3.62–10.5, *P* < 0.001) and thoracic injuries (OR 5.38, 95% CI 3.37–8.67, *P* < 0.001) were associated with increased odds of ICU admission, whereas head injuries (OR 3.21, 95% CI 1.41–7.31, *P* < 0.01) and cervical injuries (OR 3.37, 95% CI 1.08–10.5, *P* < 0.05) were associated with increased odds of in-hospital mortality.

**Table 3.** Association between increasing age and clinical outcomes.

	Count, no. (%)	Crude OR (95% CI)	Adjusted OR (95% CI)
**ICU admissions**			
65–74	38 (23.9%)	1 [Reference]	1 [Reference]
75–84	45 (21.1%)	0.85 (0.52–1.40)	0.78 (0.45–1.35)
85+	51 (19.2%)	0.75 (0.47–1.22)	0.72 (0.42–1.22)
**Mortality** ^1^			
65–74	3 (1.89%)	1 [Reference]	1 [Reference]
75–84	14 (6.57%)	3.65 (1.03–13.0)	3.46 (0.97–12.4)
85+	14 (5.26%)	2.89 (0.82–10.2)	2.83 (0.80–10.0)

1 In-hospital, all-cause mortality.

For ICU admissions: adjusted for total comorbidities, head injuries, thoracic injuries.

For mortality: adjusted for total comorbidities, head injuries, cervical injuries.

*OR*, odds ratio; *CI*, confidence interval; *ICU*, intensive care unit.

### Inter-rater Reliability

Among the 10% of the health records reviewed by a co-author, we found that our raters agreed on 88% of the information abstracted from the records, resulting in a Cohen kappa coefficient of 0.8.

## DISCUSSION

In our study population, injuries sustained after a GLF were broadly consistent among all age groups. We found that a substantial minority of older patients who underwent whole-body CT at the discretion of the treating physicians after a GLF were found to have clinically significant injuries that resulted in ICU admissions. Increasing age was not associated with an increased rate of ICU admission or death after a GLF. Thoracic injuries and ICH were associated with increased odds of ICU admission. Intracranial hemorrhages and cervical fractures were associated with increased odds of in-hospital mortality.

We found that over 30% of the injuries sustained were either ICH or thoracic injuries, both of which were associated with increased odds of ICU admissions in our study population. Intracranial hemorrhages and rib fractures among older patients are injuries that can result in high mortality rates,^8^ ^–^ ^10^ thus requiring frequent monitoring and necessitating ICU level of care. Our findings reinforce the importance of using CT to identify these injuries in patients presenting with GLFs, particularly when there is clinical suspicion of those injuries.

Furthermore, our study did not show any significant association between increasing age and ICU admission and mortality. This contrasts with the results of a previous study, which showed a stepwise increment in the rate of cervical spine injuries and in-hospital mortality associated with increased age in GLF patients from an institutional trauma registry.^11^ Patients recorded in a trauma registry will likely have sustained injuries requiring trauma team evaluation. In addition, we did not find any difference in the rates of various injuries after a GLF between the different age groups. Our study differs in that it included all patients who sustained significant injuries and those who did not. Furthermore, we included only patients who underwent whole-body CT at the discretion of the treating physicians after a GLF. Based on our findings, one should be cautious about using increased age as a risk factor alone to determine whether a patient warrants whole-body CT without considering other clinical factors.

Overall, our rates of different injuries are higher than reported in the literature. Our findings on the rate of ICH after a GLF was 12.5%, whereas the rates of ICH after a GLF reported in the literature have ranged from 3.5–7%.^12^ ^–^ ^15^ A larger, nationally representative retrospective study found the rates of thoracic and lumbar spine injuries were 1.6% and 2.5%, compared to 9.4% and 8.0% in our study, respectively.^16^ The discrepancy likely resulted from the fact that we included only patients who were selected by treating physicians to undergo whole-body CT in the ED. The treating physicians probably deem patients undergoing whole-body CT after trauma to have sustained a greater number of significant injuries during the initial evaluation.

We found that the rate of intra-abdominal injuries was low. This conclusion is broadly consistent with the literature.^11^ ^,^ ^17^ ^,^ ^18^ Of the five patients found to have intra-abdominal injuries in our study, all five were found to have either unstable vital signs, abnormal GCS, or abnormal physical exams on initial evaluation. This finding is consistent with the literature where hemodynamically stable patients with normal physical exams are unlikely to have intra-abdominal injuries after a GLF.^18^ ^–^ ^20^ Performing fewer abdominal CT scans in this population could have substantial cost savings without reducing diagnostic accuracy. Given the small number of patients who sustained intra-abdominal injuries in our study, we were not adequately powered to identify potential risk factors associated with intra-abdominal injuries after a GLF. Future prospective studies are needed to identify factors associated with intra-abdominal injuries and determine the cost-effectiveness of a selective imaging algorithm in low-risk GLF patients.

## LIMITATIONS

Our study was retrospective and susceptible to biases. Non-differential misclassification can occur during the querying of health records, which will likely bias the results toward the null. However, 10% of the health records were reviewed by co-author GS to limit this bias. Furthermore, we demonstrated excellent inter-rater reliability. The study was also susceptible to selection bias, and was limited to patients who received a whole-body CT. At our institution, the decision to order a whole-body CT on a trauma patient depends on the treating physician’s preference. Patients who received a whole-body CT are likely deemed by the treating physician to have sustained significant injuries. Therefore, our study likely overestimates injury incidence due to the selective nature of whole-body CT use. Moreover, we did not obtain information on older patients who sustained a GLF and did not receive a whole-body CT. Those two limitations likely resulted in our study overestimating the incidence proportion of injuries reported after a GLF. Furthermore, this study was based on a single institution at a tertiary-care Level I trauma center; thus, it cannot be generalized to other institutions. In addition, the nature of our query did not allow us to obtain Injury Severity Scores.

## CONCLUSION

Among patients ≥65 years of age who presented to the ED after a ground-level fall and underwent whole-body CT, thoracic injuries and intracranial hemorrhages—while a minority of the injuries sustained—were associated with increased odds of ICU admissions. These findings highlight the importance of carefully assessing these injuries in older adults. Interestingly, we found no significant differences in injury rates or clinical outcomes across age groups, suggesting that age alone should not be the determining factor for ICU admission or mortality risk in this population. Given our findings, we propose that there may be value in reassessing trauma screening protocols, especially regarding the use of whole-body CT in patients who sustain a low-energy fall. Its use should be selective rather than applied universally. Multicenter prospective studies are needed to determine the broader utility and cost-effectiveness of whole-body CT use among older patients who present to the ED after sustaining a ground-level fall.
